# Diptheria—Its Nature and Treatment

**Published:** 1879-02

**Authors:** 


### BIBLIOGRAPHICAL.

---

Diphtheria: Its nature and treatment, varieties and local expressions, By Morell Mackenzie, M.D., London, Senior Physician to the Hospital for diseases of the throat and chest, etc., etc. Philadelphia: Lindsay & Blakiston. 1879.

This monograph of a hundred pages gives the author's definition and history of the disease, and his opinion of its etiology, symptoms, diagnosis, pathology, prognosis and treatment.

While nothing new is claimed in the means proposed for the cure of this fearful malady, the various general and local remedies that have been used from time to time, are mentioned, and the probable benefit to be expected from them.

The disease is one about which physicians feel great concern, and seek the experience of others in its treatment. The book will, therefore, be read with interest by the general practitioner.

A Practical Manual of the diseases of children, with a formulary. By Edward Ellis, M.D., late senior physician to the Victoria Hospital for sick children, etc., etc. Third edition. New York: William Wood & Co., 27 Great Jones street. 1879.

This is the second issue of "Wood's library of Standard Medical Authors."

The book of two hundred and ten pages gives general rules for the dietic management of children, and describes diseases to which they are subject, with the treatment necessary for their cure. The work will be found a valuable addition to the library of physicians.

We are in receipt of two new Medical Journals, which we welcome to our exchange list: The St. Louis *Courier of Medicine and Collateral Sciences;* and the *Archives of Medicine*, published in Baltimore.

